# Cell-permeable lanthanide–platinum(iv) anti-cancer prodrugs†

**DOI:** 10.1039/d1dt01688a

**Published:** 2021-05-28

**Authors:** Kezi Yao, Gogulan Karunanithy, Alison Howarth, Philip Holdship, Amber L. Thompson, Kirsten E. Christensen, Andrew J. Baldwin, Stephen Faulkner, Nicola J. Farrer

**Affiliations:** Chemistry Research Laboratory, University of Oxford Mansfield Road OX1 3TA UK Nicola.Farrer@chem.ox.ac.uk; Department of Earth Sciences, University of Oxford OX1 3AN UK

## Abstract

Platinum compounds are a vital part of our anti-cancer arsenal, and determining the location and speciation of platinum compounds is crucial. We have synthesised a lanthanide complex bearing a salicylic group (Ln = Gd, Eu) which demonstrates excellent cellular accumulation and minimal cytotoxicity. Derivatisation enabled access to bimetallic lanthanide–platinum(ii) and lanthanide–platinum(iv) complexes. Luminescence from the europium–platinum(iv) system was quenched, and reduction to platinum(ii) with ascorbic acid resulted in a “switch-on” luminescence enhancement. We used diffusion-based ^1^H NMR spectroscopic methods to quantify cellular accumulation. The gadolinium–platinum(ii) and gadolinium–platinum(iv) complexes demonstrated appreciable cytotoxicity. A longer delay following incubation before cytotoxicity was observed for the gadolinium–platinum(iv) compared to the gadolinium–platinum(ii) complex. Functionalisation with octanoate ligands resulted in enhanced cellular accumulation and an even greater latency in cytotoxicity.

## Abstract 2

Heterometallic Eu/Gd Pt(iv) complexes have been synthesised and characterised. The Eu–Pt(iv) complex shows a luminescent “switch-on” effect upon reduction.

## Graphical abstract

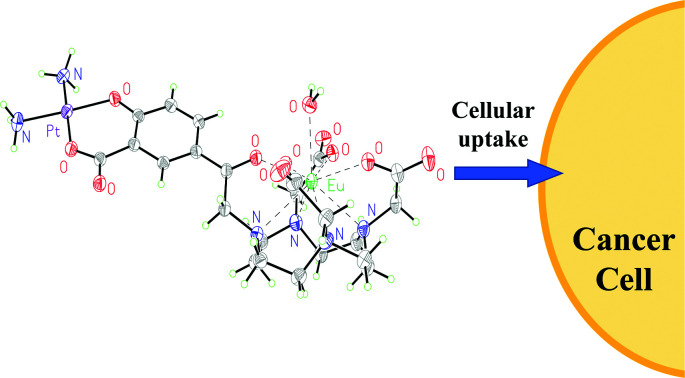

## Introduction

Approximately 50% of chemotherapy regimens worldwide include a platinum-based drug^1^ and platinum(ii) complexes such as cisplatin, carboplatin and oxaliplatin are well-established, highly effective anti-cancer agents. These complexes are highly reactive *in vivo* and the side-effects of treatment caused by off-target reactivity are often debilitating.^2,3^ Platinum(iv) complexes can potentially reduce the side-effects of treatment,^4^ as they typically require reduction to platinum(ii) species before exerting their anti-cancer effect.^5^ Their cellular uptake and rate of reduction inside cells depends strongly on the coordinated ligands.^6^ For example, lipophilic ligands such as octanoate (OA) enable [Pt^IV^(NH_3_)_2_(Cl_2_)(OA)_2_] to exhibit a 106-fold enhancement in cellular accumulation in ovarian (A2780) cancer cells in comparison to its synthetic precursor, *cis*,*cis*,*trans*-[Pt^IV^(NH_3_)_2_(Cl_2_)(OH)_2_].^7^ The additional cellular uptake for the octanoate complex results in an increased cytotoxicity by two orders of magnitude, compared to cisplatin.^8^

Lanthanide complexes have played a central role in the development of magnetic resonance imaging (MRI) agents for over thirty years.^9–11^ Gadolinium complexes with octadentate ligands have favourable relaxation properties that have enabled them to become a key tool in diagnostic imaging.^12^ While initially employed for imaging vasculature,^13^ through careful ligand design “smart” systems have been developed that can be targeted to particular tissue types, or that exhibit a response modulated by external biochemical stimuli.^14,15^ To enable us to localise gadolinium compounds, we recently developed a diffusion-based ^1^H NMR method that can be used to distinguish between intra- and extracellular pools of water, relying on the fact that water diffusion in intracellular fluids is defined by the boundaries of the cell.^16^ Since the cell is much smaller than the voxel (volume pixel) in an MRI image, this means that intracellular fluid can be treated as a slow diffusing pool, whilst extra-cellular fluid is treated as a fast diffusing pool. The INDIANA (IN cell DIffusion Analysis) methodology allows these two pools and their properties to be quantitatively described, including relaxation rates.

Complexes which incorporate lanthanides such as europium also offer attractive luminescence properties. The general concept of generating “switch-on” luminescence following reduction of a platinum(iv) prodrug to platinum(ii) is a highly promising strategy for tracking (sub)cellular distribution and reduction of platinum(iv) prodrugs. Previously reported strategies include axial platinum(iv) coordination of quenched fluorophores such as fluorescein^17^ and selective fluorescence reporting from organic probes following reaction with specific platinum(ii) reduction fragments.^18^ Lanthanides provide a significant advantage over purely organic fluorophores, by producing a highly distinctive emission profile when excited, from which background autofluorescence can also be effectively removed through time-gating.^19^

In order to develop redox-active platinum anti-cancer agents, whilst simultaneously providing a means to accurately track their distribution in the body, two gadolinium(iii)–Pt(iv) complexes derived from carboplatin and cisplatin have been recently developed.^20^ Moreover, a series of platinum(iv) prodrugs conjugated to Gd-texaphyrin have shown greater stability towards hydrolysis and nucleophilic attack compared to their platinum(ii) analogues, while the Gd-texaphyrin fragment presented the ability to activate the platinum(iv) prodrug species through redox cycling.^21^

Microscopy on luminescent analogues can provide insights in isolated cells or tissue slices,^22,23^ but does not translate readily to whole body imaging. Similarly, MRI resolution does not approach the diameter of a single cell, meaning that it is difficult to assess distribution of a contrast agent between intra- and extra-cellular fluid. In this manuscript, we address the issue of how MR methods can be used to define cellular uptake by exploiting the constraints on diffusion imposed by intracellular localization of a bimetallic lanthanide–platinum complex.

Drawing these concepts together, we have synthesized and characterized a dual-purpose lanthanide(iii)–platinum(iv) system which includes a sensitizing salicylic group. This can enable both *in cellulo* tracking in preclinical development (Ln = Eu, Gd), and *in vivo* tracking (Ln = Gd), through simple modulation of the lanthanide, whilst retaining very similar pharmacological properties regardless of the choice of lanthanide. This first reported example of a Eu(iii)–Pt(iv) complex that has the potential to provide highly diagnostic real-time.

**Fig. 1:**
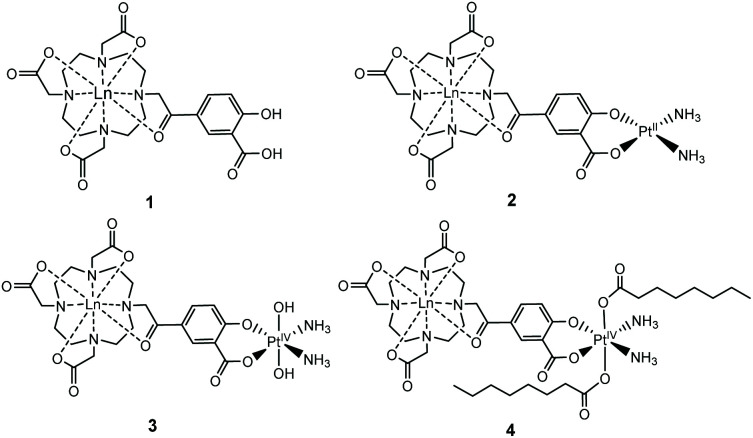
Structures of the complexes **1–4** reported, where Ln = Gd, Eu.

We established the minimal cytotoxicity of the gadolinium salicylic acid precursor, and the contrasting cytotoxicity of the Gd(iii)–Pt systems, and correlated accumulation with the effect that oxidation state and choice of axial ligand of the platinum group has on the cytotoxicity of these complexes. Information on the reductive activation of a Pt(iv) prodrug. Our established diffusion-based ^1^H NMR method enabled us to determine the extent to which the new complexes **1–4** (Fig. 1) accumulated within cells.

## Results and discussion

**Scheme 1:**
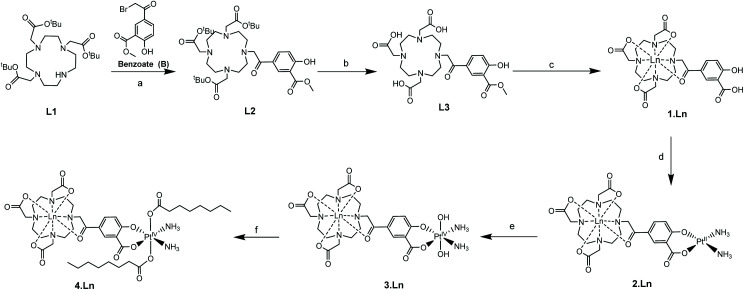
Synthetic route to the complexes (Ln = Gd, Eu). Reagents and conditions: (a) K_2_CO_3_, MeCN, room temperature, 16 h; (b) TFA, CH_2_Cl_2_, 24 h; (c) Ln(OTf)_3_, MeOH, 60 °C, 48 h; (d) Pt(NH_3_)_2_I_2_, AgNO_3_, H_2_O_2_, pH 10, room temperature, 24 h; (e) H_2_O_2_, H_2_O, room temperature, 16 h; (f) octanoyl chloride, acetone, pyridine, room temperature, 16 h.

We have previously used phenacyl–DO3A derivatives to good effect, as kinetically stable building blocks for more complicated architectures.^24–26^ The lanthanide complex (**1**) (Fig. 1) (where Ln = Gd, Eu) builds on this work, and was conceived as a system in which the lanthanide is bound in an octadentate binding site, while the pendent salicylic acid group can act as a ligand to a second metal centre – such as platinum – to give **2**. The synthesis of the ligands and complexes are summarized in Scheme 1. Triester (**L1**)^27^ was reacted with methyl 5-bromoacetyl-2-hydroxybenzoate (**B**), to yield the protected pro-ligand, **L2**. Deprotection was accomplished by treating **L2** with TFA to give the unmasked pro-ligand, **L3**, which was reacted with the appropriate lanthanide trifluoromethane sulfonate to produce the desired Ln(iii)–Pt(ii) complex **1·Ln** (where Ln = Gd, Eu). Reaction of **1·Ln** with *cis*-[Pt(NH_3_)_2_(OH_2_)_2_]^2+^ yielded the heterometallic complex **2·Ln**.^28^ Oxidation of **2·Ln** with H_2_O_2_ produced the Ln(iii)–Pt(iv) di-hydroxido complex **3·Ln**.^29^ Reaction of **3·Ln** with octanoyl chloride afforded the Ln(iii)–Pt(iv) di-octanoic acid conjugate, **4·Ln**.^8^ All compounds were purified by LCMS, with characterisation data consistent with the proposed structures (see ESI†) and in accordance with previously reported Ln(iii)–Pt(ii) complexes.^30^ The complexes were also characterized by ICP-MS to confirm the Ln : Pt ratio and ensure that complexation had occurred (Table S1†).

**Fig. 2:**
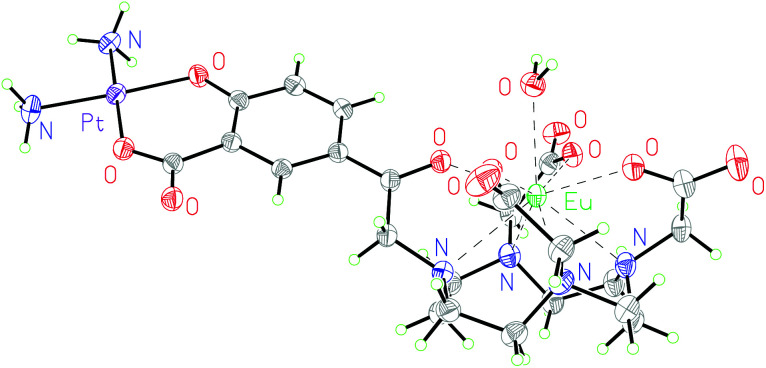
Molecular structure of **2·Eu** from single crystal X-ray diffraction studies; thermal ellipsoids are drawn at 20% probability and water in the solvent sphere is omitted for clarity.

Additionally, complex **2·Eu** was crystallised from aqueous solution at 4 °C and characterised by single crystal X-ray diffraction. The structure was solved by charge-flipping using ‘Superflip’,^31^ and refined by full-matrix least squares on *F*^2^ using CRYSTALS suite (Fig. 2).^32–34^ The structure demonstrated a monocapped square antiprism (SAP) configuration of the Eu centre.

To the best of our knowledge, this is the first reported europium–platinum X-ray single crystal structure with a kinetically stable macrocyclic lanthanide complex; bond lengths and angles are essentially consistent with other previously reported Ln(iii)–Pt(ii) complexes.^35,36^

**Fig. 3:**
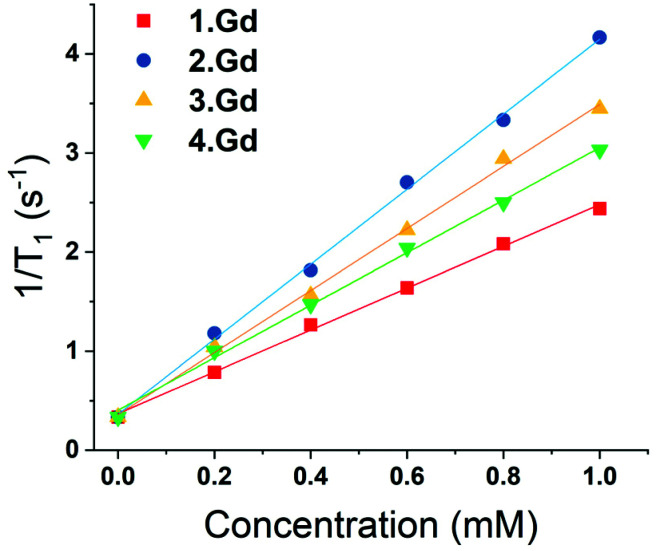
Variation of 1/*T*_1_ with concentration of gadolinium for complexes **(1–4)·Gd** at different concentrations (at 25 °C, in DMEM, 14.1 T).

Complexes **1–4** were purified by HPLC with different retention times (see Fig. S13† for the HPLC traces of **(1–4)·Gd** complexes). The complexes were also characterised by mass spectroscopy and NMR spectroscopy. ^195^Pt NMR spectroscopy of **2·Lu** revealed a single resonance at −1608 ppm (D_2_O, Fig. S8†), while **3·Lu** gave a resonance at 1691 ppm (D_2_O, Fig. S9†), which are consistent with Pt(ii) and Pt(iv) oxidation states, respectively.^37^ Whilst the Pt(ii) species **2·Lu** was observed as a singlet, the Pt(iv) complex **3·Lu** was observed as a quintet, as a result of coupling to the two equivalent quadrupolar (*I* = 1) ^14^N nuclei of the NH_3_ ligands. The magnitude of the ^1^*J* (^195^Pt, ^14^N) coupling constant (213 Hz) is consistent with other ^1^*J* (^14^N, ^195^Pt) constants that we have previously observed, for example in the platinum(iv) species, *cis*,*cis*,*cis*-[Pt(N_3_)_2_(OH)_2_(NH_3_)_2_] (167 Hz).^38^ The ^1^H NMR spectra of the europium complexes (Fig. S10†) are consistent with those of other phenacyl–DO3A analogues, and show structures dominated by the square antiprismatic diastereoisomers of the complexes, in agreement with the molecular structure of **2·Eu** determined using single crystal X-ray diffraction. Measurement of the *T*_1_ relaxation enhancements obtained with complexes **(1–4)·Gd** offers further insights into their solution state structure. The relaxivity of the complexes was determined by observing the variation of 1/*T*_1_ with complex concentration in media (DMEM) as shown in Fig. 3. In all cases, the resulting plots showed a linear response to concentration across a wide range, indicating that all complexes were essentially stable in solution, and that their properties are not changed by their local environment (since a change in speciation would be expected to result in curvature of the plots). All the complexes **(1–4)·Gd** exhibit relaxivities consistent with a single water molecule in their inner coordination sphere (Table 1).

**Table 1:** Relaxivities of complexes **1·Gd–4·Gd**

Compound	Relaxivity (s^−1^ mM^−1^)	(±)
**1·Gd**	2.21	0.04
**2·Gd**	3.96	0.12
**3·Gd**	3.21	0.1
**4·Gd**	2.89	0.11

This is corroborated by the molecular structure of the europium analogue, **2·Eu**, determined using single crystal X-ray diffraction. This shows a single bound water molecule in the inner coordination sphere of the lanthanide (Fig. 2). **1·Eu** displays luminescence properties in line with other phenacyl–DO3A derivatives, exhibiting intense lanthanide-centred emission. The luminescence lifetimes (*τ*_H_2_O_ = 0.66 ms and, *τ*_D_2_O_ = 2.13 ms, Table S3†) were used to calculate the number of inner sphere solvent molecules using established methods.^39^ The value obtained (*q* = 1.0) is consistent with expectations for a europium ion complexed by an octadentate ligand. **2·Eu** displays very similar luminescence properties: the observed luminescence lifetimes (*τ*_H_2_O_ = 0.61 ms, *τ*_D_2_O_ = 1.87 ms) are also consistent with *q* = 1.0 and with the structure of **2·Eu** determined from X-ray diffraction studies.

**Fig. 4:**
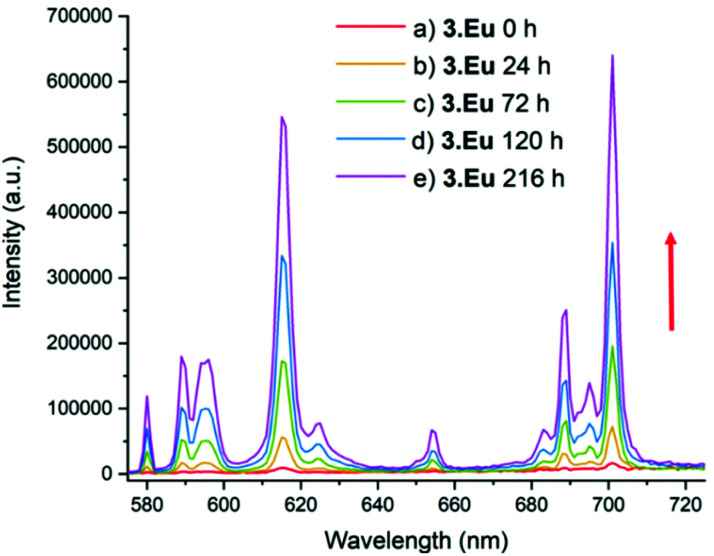
Luminescence spectrum of **3·Eu**; *λ*_ex_ = 330 nm, emission slit = 1 nm. (a) before addition, (b) 24 h after addition of excess (20-fold) ascorbic acid (AA) and (c) 72 h after addition of AA, (d) 120 h after addition of AA, (e) 216 h after addition of AA. The red arrow shows that as time increases the intensity of emission increases.

Furthermore, the steady state spectra of the two complexes show strong similarities, suggesting that the coordination environments at the europium ion are similar. In comparison, **3·Eu** and **4·Eu** exhibited only very weak luminescence, and poor signal-to-noise ratios were obtained (Fig. S15†). However, when the platinum(iv) centre in **3·Eu** was treated with ascorbic acid (AA), a dramatic enhancement in luminescence intensity was observed – resulting in a 37-fold enhancement of signal on regenerating **2·Eu** (Fig. 4). The excited-state manifold of the platinum(iv) complex acts to quench the europium emission, meaning that switching on of signal can potentially be used to observe reduction of the complex *in situ*. The bis-octanoic acid platinum(iv) complex **4·Eu** also showed a “switch-on” in luminescence when reduced with ascorbic acid (Fig. S16†).

This is consistent with other Pt(iv)/Pt(ii) couples that display reductive “switch on” fluorescence; the quenching in the Pt(iv) state being thought to arise from the presence of metal-based d-orbitals energetically positioned between the HOMO and LUMO of the fluorophore.^40^ Quenching of the Eu(iii) emission by the Pt(iv) centre is therefore suggested to occur through energy transfer to low-lying electronic states in the platinum(iv) complex. Luminescence in the Pt(iv) oxidation state is more likely to be observed if one or more ligands are cyclometallated.^41,42^ Although the mechanism of reduction *in cellulo* is likely to be complicated and involve more than one reducing species,^43^ ascorbate acid-mediated reduction gives an initial insight into the potential for monitoring the reduction of these compounds through fluorescence microscopy. During cellular processing it is anticipated that the lanthanide–salicylic acid ligand will dissociate from the platinum(ii) complex which is likely to further affect the lanthanide luminescence, further study is needed to investigate this modulation in detail.

As shown previously, using the INDIANA model,^16^ the diffusive behaviour of water within cellular systems can be robustly described using a two-pool model with the water residing in either an intra- or extra-cellular pool. This results from the intrinsic diffusion of water being slower in the viscous intra-cellular compartment and its maximum displacement being restricted by the presence of the cell membrane. The model also explicitly accounts for exchange between the two environments. This methodology builds on previous experiments to characterise restricted diffusion in cellular systems.^44–47^

**Fig. 5:**
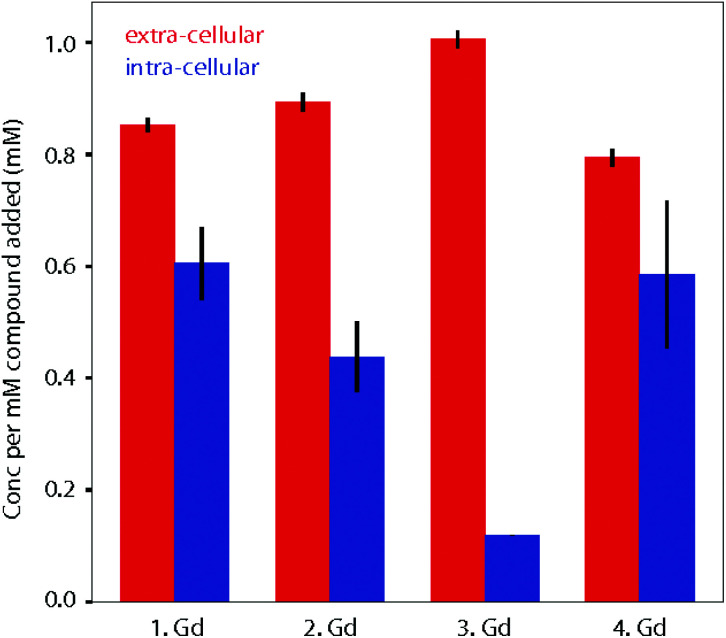
Calculated partitioning of complexes **1–4·Gd** between extra- and intra-cellular environments following 1.5 h incubation with KNS42 cells. The concentration of complexes in the two environments is found by comparing longitudinal relaxation rates in the presence and absence of the compounds (Δ*R*_1_) and then dividing by the relaxivity. The error bars are calculated by propagating the error in Δ*R*_1_ for three independent samples and taking the standard deviation.

When fitting variable diffusion delay data from ^1^H NMR spectroscopic experiments, it is possible to derive a number of system properties including intra- and extra-cellular diffusion coefficients, populations of water in the two environments, exchange rate of water over the membrane, average cell radius and, crucially for this study, intra- and extra-cellular longitudinal relaxation rates of water. Due to the relaxation enhancement caused by MRI imaging reagents, by comparing the difference in relaxation rates in the presence and absence of these agents in cellular systems we can quantitatively assess the degree to which they are localised within cells. As the relaxivities of the four complexes are different, this must be considered when using an increased intra-cellular relaxation rate as a proxy for cellular uptake of imaging agent. To address this, we used the relaxivity of the complexes measured in DMEM (Fig. 3). To estimate the partitioning of the four complexes between extra- and intra-cellular environments we assumed that the relaxivity of the compounds inside cells could be approximated by their relaxivity in media. Using the INDIANA method described above we found the extra- and intra-cellular longitudinal relaxation and compared this to the values in the absence of any complex to give Δ*R*_1_ for each compound in the two environments. This value was then divided by the relaxivity to show how the compound was partitioned. The results of our analysis are shown in Fig. 5. The ^1^H diffusion measurements were used to quantify the accumulation of the compounds within cells. Complex **1·Gd** accumulated relatively well in the cells, whilst there was a small drop reduction in accumulation for **2·Gd** which incorporated the Pt(ii) group. Gadolinium complexes enter cells predominantly by micropinocytosis,^48,49^ whereas platinum(ii) complexes enter cells through a combination of copper transporters, anion/cation transporters and passive diffusion.^50^ Significantly lower cellular accumulation was observed for the platinum(iv) compound, **3·Gd**, consistent with the oxidation state and ligand sphere of the Pt modulating the ability of the complex to enter cells. The copper transporter mechanism is less viable for platinum(iv) complexes, since there are no vacant positions available in the octahedral platinum(iv) coordination sphere. Cellular uptake of platinum(iv) complexes is therefore thought to occur through a combination of passive diffusion and active pathways.^51^

Finally, **4·Gd**, which incorporates OA ligands, showed a similar uptake to **1·Gd**, demonstrating the notable effect of the modified coordination sphere of the platinum(iv) on the degree of cellular accumulation. If diffusion is the predominant cellular uptake of the mechanism for **3·Gd**, increasing the lipophilicity through inclusion of octanoic acid groups as in **4·Gd** would be anticipated to significantly enhance uptake, and the data are consistent with this, although without discounting the possibility of uptake through micropinocytosis. The dominant route(s) of cellular uptake for other lanthanide–platinum conjugates are unknown, since only a handful of examples have been reported, with either labile,^52,53^ or kinetically inert lanthanide chelation.^19,20,27^ From these data, we suggest that the route(s) vary significantly depending on the coordination sphere platinum. These diffusion results are particularly enlightening in combination with the cytotoxicity results to give an overall picture of the cellular behaviour of the complexes.

A paediatric glioma cell line was specifically chosen for investigation, due to our interest in developing novel anti-cancer compounds for these malignancies.^54^ Complex **1·Gd** was well-tolerated by KNS42 (paediatric glioblastoma) cells (viability <90%) across all concentrations and time-points tested. This indicated minimal cytotoxicity for **1·Gd**, with IC_50_ values exceeding 1000 μM (Table 2, Table S5, Fig. S18†). The Gd(iii)–Pt(ii) complex **2·Gd** demonstrated increasing cytotoxicity after 1.5 h exposure (IC_50_ = 637.9 μM at 1.5 h incubation, Fig. S18†). The Gd(iii)–Pt(iv) complex **3·Gd** was significantly more cytotoxic than complex **2·Gd** after 1.5 h (*P* < 0.0001) and 24 h (*P* = 0.0031) exposure. However, no significant difference in cytotoxic effect between **2·Gd** and **3·Gd** was observed at longer (48 h and 72 h) exposure times (*P* values 0.0584 and 0.1482 respectively) (Fig. S18†). Complex **4·Gd** was less cytotoxic than **2·Gd** and **3·Gd** at all exposure times, with IC_50_ > 1000 μM at 1.5 h and 24 h, with increasing cytotoxicity after 48 h exposure (*P* = 0.0027 and <0.0001) (Fig. S18†). Whilst complex **1·Gd** showed no toxicity towards cells up to a concentration of 1000 μM, the platinum-containing bimetallic complexes **2–4·Gd** displayed varying degrees of cytotoxicity. As kinetically-inert 5d^6^ complexes, platinum(iv) complexes are suggested to reduce to platinum(ii) intracellularly before ligand exchange and binding to cellular targets (*e.g.* DNA, proteins) can occur.^55^ Complexes **3–4·Gd** all demonstrated increased cytotoxicity towards the cells with prolonged contact time. However, complex **4·Gd** showed a more pronounced “latent” period of initial lower cytotoxicity, compared with complexes **2·Gd** and **3·Gd**, consistent with an outer-sphere mechanism of reduction for **4·Gd** which is likely to be slower than an inner-sphere mechanism of reduction for **3·Gd**. For **3·Gd**, the reduction mechanism could involve bridging to one or both axial hydroxido groups,^5^ which is not possible for **4·Gd**. An investigation into the sub-cellular location and time-resolved fluorescence reporting of complexes **1·Eu–4·Eu** using microscopy is planned.

**Table 2:** Cytotoxicity (IC_50_, μM) values of compounds **(1–4)·Gd** in KNS42 cell line. Cells were exposed to ½ log serial dilutions of compounds for 1.5, 24, 48 and 72 h. IC_50_ values were determined using a 3 parameter log inhibitor *vs.* concentration curve fit. Two independent sets of three (6 in total) were performed for all compounds. Mean IC_50_ values and 95% confidence interval (CI) are shown below; *P* values are given in the ESI (Table S4†)

Complex	**1·Gd**		**2·Gd**		**3·Gd**		**4·Gd**	
Time/h	IC_50_	95% CI	IC_50_	95% CI	IC_50_	95% CI	IC_50_	95% CI
1.5	>1000	—	637.9	446.1–934.3	227.6	183.2–283.7	>1000	—
24	>1000	—	316.7	226.6–445.9	96.5	80.13–116.4	>1000	—
48	>1000	—	75.3	65.79–86.25	91.4	73.43–114.0	747.5	644.4–871.2
72	>1000	—	75.7	64.68–88.82	53.2	43.34–65.50	142.8	111.2–84.5

## Conclusions

Complexes **(1–4)·Gd** all show cellular accumulation in paediatric glioblastoma (KNS42) cells, as demonstrated by diffusion ^1^H NMR spectroscopic experiments. At equimolar concentrations, the platinum(iv) complex **3·Gd** showed significantly lower cellular accumulation than the platinum(ii) complex **2·Gd** after 1.5 h. Complex **3·Gd** was derivatised through inclusion of octanoate axial ligands to produce **4·Gd** which enhanced the cellular accumulation of the complex in comparison to **3·Gd**. The lack of cellular toxicity of **1·Gd** suggests that lanthanide complexes based on **1** have significant potential for development as cell-permeant probes. The cellular accumulation, cytotoxicity and “switch-on” luminescence following reduction of prodrugs **3·Ln** and **4·Ln** makes them ideal candidates for studying the real-time accumulation and reduction of platinum(iv) prodrugs *in cellulo*, as well as making them candidates for further development as anti-cancer prodrugs. ^1^H NMR diffusion-based models have considerable potential in establishing compound localisation and internalization and open up a new approach for using magnetic resonance imaging modalities to achieve this goal *in vivo*.

## Author contributions

SF and NF and AB were responsible for conceptualization, formal analysis, funding acquisition, methodology, project administration, resources, supervision, visualization and writing. KY carried out synthesis and characterisation, GK and KY carried out diffusion NMR experiments. AH carried out the biological experiments with assistance from KY. PH conducted ICP-MS experiments. ALT and KEC solved the X-ray crystallographic structure.

## Conflicts of interest

There are no conflicts to declare.

## Supplementary Material

DT-050-D1DT01688A-s001

DT-050-D1DT01688A-s002
