# Supplementary material for: Cell-permeable lanthanide–platinum(iv) anti-cancer prodrugs
Source: Dalton Trans. 2021 May 28;50(25):8761–7. doi: 10.1039/d1dt01688a (PMC8237448; doi:10.1039/d1dt01688a)
Supplement: DT-050-D1DT01688A-s001 [file DT-050-D1DT01688A-s001.pdf]

Electronic Supplementary Material (ESI) for Dalton Transactions.
This journal is © The Royal Society of Chemistry 2021

Electronic Supplementary Information for

Cell-Permeable Lanthanide-Platinum(IV) Anti-Cancer Prodrugs

Kezi Yao, Gogulan Karunanithy, Alison Howarth, Philip Holdship, Amber L. Thompson, Kirsten E. Christensen, Andrew J. Baldwin, Stephen Faulkner, Nicola J. Farrer.

Table of Contents

Experimental Procedures 1
Materials and methods 1
Synthesis 4
Cell culture for NMR In-cell diffusion 10
NMR spectra 11
HRMS 13
HPLC 15
Single Crystal X-ray Diffraction Studies 16
Relaxivity 34
UV spectra 35
Excitation and Emission Spectra 36
Lifetimes 38
Cytotoxicity Experiments 39
References 40

Experimental Procedures

Materials and methods

Chemical reagents:

$K_2[PtCl_4]$ was purchased from Precious Metals Online. HPLC-grade solvents and Millipore-filtered H_2O were used for the preparation of compounds and purification by HPLC. All other chemical reagents were purchased from standard commercial vendors (*e.g.* Sigma-Aldrich or Alfa Aesar) and used as received. (IM) indicates use of a nylon syringe filter (pore size 0.2 µM).

NMR spectroscopy (Characterisation):

Spectra were acquired at 298 K unless otherwise stated, and processed using Topspin 3.2. All chemical shift (δ) values are given in parts per million and are referenced to residual solvent unless otherwise stated, J values are quoted in Hz.

1D ^{1}H NMR spectra: spectra were acquired on a Bruker AVIIIHD 500 MHz (500.13 MHz) equipped with a 5mm z-gradient broadband X-^{19}F/^{1}H BBFO SMART probe or a Bruker AVIIIHD 400 nanobay (400.17 MHz).

1D ^{13}C NMR and ^{195}Pt NMR spectra: spectra were acquired on a Bruker AVII 500 MHz spectrometer equipped with a z-gradient triple resonance inverse $^{1}H/^{19}F(^{13}C)$ TXI probe. ^{195}Pt chemical shifts were externally referenced to K_2PtCl_6 in 1.5 mM HCl in D_2O (δ 0 ppm).

NMR spectroscopy cell sample preparation:
Cell samples were concentrated to 250 µL and transferred to 5 mm Shigemi NMR tubes to ensure that the cells remained within the active coil volume for the duration of the experiment. 5% D_2O was added to all samples to provide a lock signal.

NMR Spectroscopy (Diffusion Experiments):
All diffusion NMR experiments were acquired at 298 K on a 14.1 T (600 MHz) Agilent DD2 NMR spectrometer equipped with a 5 mm z-axis gradient triple resonance room temperature probe. Gradients were calibrated using the known diffusion coefficient of residual HDO (1902 $\mu m^2 s^{-1}$) in a sample of D_2O at 298 K. The spectrometer temperature was calibrated with d_4-methanol. A stimulated echo pulse sequence with six variable diffusion delays (Δ = 50, 100, 200, 300, 400 and 500 ms) was used in all diffusion experiments. In all experiments δ= 2 ms, τ2, the time for which the magnetisation is transverse, was always set to 10 ms and eleven quadratically spaced gradient field strengths between 8 and 60 $G \cdot cm^{-1}$ were employed.

All spectra were Fourier transformed and phased using NMRPipe. Data were analysed in phase-sensitive mode, with the lowest gradient field strength and diffusion delay spectrum used to determine the phase parameters that are then applied to all other spectra. The water peak intensity in each spectrum was integrated and analysed using the INDIANA method[1] to determine the intra-cellular water mole fraction, cell radius, water exchange rate over the membrane and diffusion coefficients and longitudinal relaxation rates in both the intra- and extra-cellular spaces. Previous simulations have shown that while the INDIANA method is relatively insensitive to small changes in the intra-cellular longitudinal relaxation rate it is sensitive to when this rate exceeds approximately 0.5 s^{-1}.

NMR Relaxivity Experiments:
Relaxivity data were acquired at 298 K on a 14.1 T (600 MHz) Agilent DD2 NMR spectrometer equipped with a 5 mm z-axis gradient triple resonance room temperature probe. In all cases longitudinal relaxation rates were measured using an inversion recovery pulse sequence. To minimise radiation damping effects 100 uL of sample was placed in a co-axial NMR tube and CDCl3 was placed in the outer tube to provide lock signal. For all experiments the recycle delay time was set to at least ten times the T1 time to ensure that the magnetisation relaxed fully back to equilibrium prior to the inversion pulse. Typically, fifteen different relaxation delays up to five times the T1 time were employed. Spectra were subsequently phased and Fourier transformed using NMRPipe. Peak intensities for each relaxation delay were integrated and fit to the equation: $S(t) = S(\infty)\left(1 - 2e^{-R_1 t}\right)$) where S is the signal intensity and $S(\infty)$ and R_1 are fitting parameters using in-house python scripts that employ the LMFIT and nmrGlue modules.

Mass Spectrometry:
Low resolution ESI-MS were obtained with a Waters Micromass LCT Premier XE spectrometer.
HRMS: Spectra obtained with a Thermofisher Exactive Plus with a Waters Acuity UPLC system.
MS/MS experiments: Spectra were performed on an Acuity UPLC in flow injection analysis mode, equipped with a Waters Xevo G25 QTOF. MS data were processed using MassLynx 4.0.

Elemental analysis (Pt, Ln) of complexes: complexes were dissolved in DMEM media and analysed by ICP-MS analysis. 5 repeat samples for each complex were prepared. The average of the replicates were calculated including error calculation. 50 µL of compound containing media was added to the PFA vials, to which 500 µL 16M HNO_3 and 100 µL of 30% H_2O_2 were added. The samples were digested overnight at 70° C. Following this digestion the samples were allowed to cool to room temperature, where they appeared transparent and yellow in colour. Following complete digestion, the samples were diluted to 15 mLwith water.
Trace elemental analyses of Pt and Gd, were undertaken by inductively coupled plasma - mass spectrometry (ICP-MS), using a PerkinElmer NexION 350D ICP-MS at the Department of Earth Sciences, University of Oxford. All sample and standard measurements were blank corrected, using blank measurements taken periodically during the analytical run. The run was arranged so that every ten samples were bracketed by a gravimetrically prepared calibrant standard, as a gauge for analytical precision and accuracy. A secondary quality control standard was also measured to verify the validity of the calibration. Detection limits are calculated as three times the standard deviation of the blank series measured during the analysis (ca. n=19) and corrected for the sample dilutions.

HPLC:
HPLC were performed with a Waters Autopurification system.
prep-HPLC: Prep-HPLC used a Waters X-Bridge OBD semi-prep column (5 µm, 19 mm x 50 mm), with an injection loop of 1 ml, eluting with H_2O+0.1% formic acid/MeOH +0.1% formic acid. Samples (in H_2O/MeOH) were filtered (IM) and injected in 750 µL aliquots, with mass-directed purification with an ACQUITY QDa performance mass spectrometer.
Analytical HPLC: Analytical HPLC used the same solvents and a Waters X-Bridge OBD column (5µm, 4.6 mm x 50 mm) and an injection loop of 0.02 ml. Retention times (tR) are quoted for the solvent gradient: 0 min (95% A : 5% B); 1 min (95:5), 7.5 min (5:95) on the analytical column.

UV-visible absorption spectra and luminescence spectra:
Spectra were obtained with a T60U Spectrometer PG Instruments Ltd using UVWin Software, or the Waters HPLC.

HORIBA Jobin Yvon FluoroLog3 fluorimeter (Hamamatsu R928 detector and a double-grating emission monochromator) was used to acquire the luminescence spectra. The standard conditions for acquiring emission and excitation spectra are room temperature and steady-stated mode unless otherwise stated. HORIBA Jobin Yvon FluoroLog3 fluorimeter system equipped with a Xenon flash lamp was used to acquire emission lifetimes. Luminescence lifetimes were obtained by tail fit for Eu(III) complexes using Origin software.

Synthesis
^{t}BuDO3AH: 1,4,7-Tris(tert-butoxycarbonylmethyl)-1,4,7,10- tetraazacyclododecane, hydrobromide salt (**L1**) was synthesized as previously reported.[2]

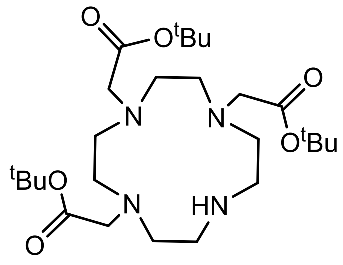

Figure S1 compound **L1**

Methyl 5-(2-bromoacetyl)-2-hydroxybenzoate (Benzoate)

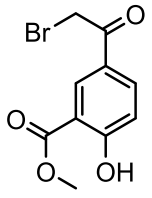

Figure S2. Benzoate

To a stirred solution of methyl 5-acetylsalicylate (1.0 g, 5.1 mmol) in $CHCl_3$/EtOAc (40 mL) was added copper (II) bromide (2.384 g, 10.8 mmol). The reaction mixture was gently refluxed at 45 °C. The progress of the reaction was monitored by TLC. After completion of the reaction, the reaction mixture was filtered and water (50 mL) and EtOAc (40 mL) were added to the reaction mixture. EtOAc layer was separated and aqueous layer was re-extracted with EtOAc (20 mL x 2). The EtOAc fractions were combined, dried over anhydrous $MgSO_4$, filtered, and the supernatant concentrated under reduced pressure to afford the crude 5-bromoacetyl-2-hydroxybenzoic acid methyl ester as a yellowish white solid. Crude product was recrystallized using CH_2Cl_2 and hexane. (1.131g, 82%) **^{1}H NMR** (400MHz, $CDCl_3$) δ: 11.33 (s, 1H), 8.52 (d, 1H), 8.11(dd,1H), 7.07(d, 1H), 4.40(s, 2H), 4.01 (s, 3H) ppm. **^{13}C NMR** (126 MHz; $CDCl_3$) δ: 189.4, 170.0, 166.0, 136.2, 132.3, 125.7, 118.6, 112.5, 53.0, 30.4. **ESI-MS** (MeOH): 272.97 [**benzoate** +H]$^{+}$.

2,2',2''-(10-(2-(4-hydroxy-3-(methoxycarbonyl)phenyl)-2-oxoethyl)-1,4,7,10-tetraazacyclododecane-1,4,7-triyl)triacetic acid (L2)

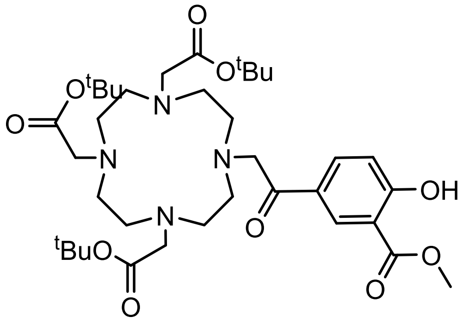

Figure S3. Compound **L2.**

To a solution of **L1**(0.772g 1.5 mmol) (*1*) in dry acetonitrile (35 ml), potassium carbonate was added (0.415g, 3 mmol) followed by the addition of **benzoate** (0.408g, 1.5mmol). The suspension was left stirring at ambient temperature under an inert (N_2) atmosphere overnight. The potassium carbonate was removed by filtration and the solvent was removed from the filtrate by reduced pressure. The crude product was purified by silica gel column with MeOH and CH_2Cl_2. The resulting product was deprotected with TFA (6 mL) in DCM (12 mL) (0.452g, 56%). **^{1}H NMR** (400MHz, D2O): 8.43 (d, 1H), 8.24(s, 1H), 8.07(dd, 1H), 3.98(s, 3H), 3.94-2.88 (br, 24H) ppm. **^{13}C NMR** (126 MHz; $CDCl_3$) δ: 169.4, 165.9, 164.0, 135.0, 131.5, 117.9, 112.7, 52.9, 48.8, 57.1-46.8(br). **ESI-MS** (MeOH): 539.23 [**2**+H]$^+$.

General procedure for the synthesis of salicylic acidDO3A lanthanide complexes (1)

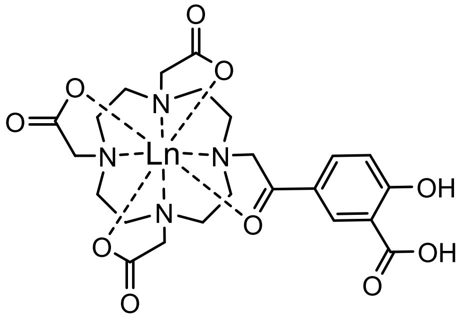

Figure S4. Compound **1.Ln**

1.1 equivalents of $Ln(OTf)_3$ was added to a solution of **L2** (100 mg, 0.19 mmaol) in MeOH (3 mL). The solution was stirred at 60 ^{o}C for 30 min, the pH adjusted to 5 using 1M NaOH aqueous solution, and the resultant solution stirred at 60 ^{o}C for 2 d. The MeOH was removed under reduced pressure, leaving an oil that was dissolved in H_2O (3 mL), and the pH was adjusted to 10 by dropwise addition of aqueous NaOH (1M) to remove excess Ln^{3+} ions precipitates by centrifuge.

The resulting supernatant was stirred at 50°C overnight. Formic acid (1M) was added to adjust pH to 7. The resulting product was purified by mass-directed HPLC and the solvent removed to give the title compound as an off-white powder.

1.Lu. Yield: 48%. **^{1}H NMR**(400MHz,D_2O): 8.59 (d, 1H), 8.14 (dd, 1H), 6.95 (d, 1H), 3.91-2.37 (br, 24H)ppm, **ESI-MS** (MeOH): 697.13 [**Lu.1**+H]$^+$.

1.Eu. Yield: 32%. **^{1}H NMR**(400MHz,D_2O): 35.05(s), 28.17(s), 27.24(s), 26.27(s), 17.08(s), 12.40(s), 9.18(s), 8.65(s), 7.27(s), -1.76(s), -3.91(s), -4.35(s), -5.41(s), -7.76(s), -8.35(s), -10.01(s), -10.26(s), -11.38(s), -

11.82(s), -12.16(s), -13.04(s), -13.38(s), -15.29(s), -16.16(s), -17.91(s)ppm. Only resolved peaks outside the 0 to 5 ppm region are reported. **ESI-MS** (MeOH): 675.11 [**1.Eu**+H]$^+$. UV-Vis (H_2O): λ_{max} 309 nm.

1.Gd. Yield: 52%. **ESI-MS** (MeOH): 680.11 [**1.Gd**+H]$^+$, UV-Vis (H_2O): λ_{max} 309 nm, **ICP-MS** Pt: Gd = 0:1

General procedure for the synthesis of salicylic acidDO3A lanthanide-platinum(II) complexes (2)

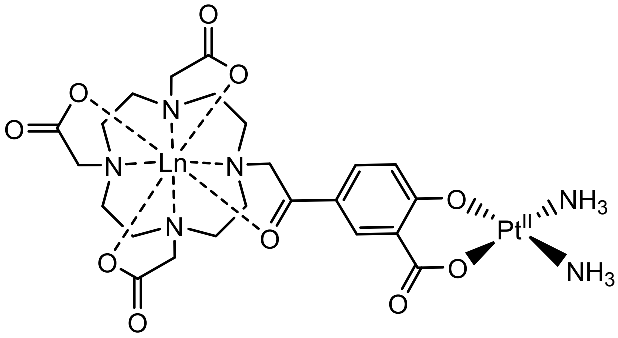

Figure S5. Compound **2.Ln**

Complex 1.Ln, 1 equivalent $PtI_2(NH_3)_2$ (73mg, 0.15 mmol) and 2 equivalent AgNO3 (51 mg, 0.30 mmol) were dissolved in 5 mL H_2O, saturated Na_2CO_3 solution was added to adjust pH to 9, stirred at room temperature overnight. The resulting grey precipitates were removed by centrifuge and the crude product was purified by HPLC in acidic condition.

2.Lu. Yield: 23%. **^{1}H NMR**(400MHz,D_2O): 8.78 (d, 1H), 7.96 (dd, 1H), 6.84 (d, 1H), 4.28-4.19 (s, 3H), 4.18-4.08 (s, 3H) 3.71-2.35 (br, 24H)ppm, **^{13}C NMR**(500MHz,D_2O): 215.31, 180.76, 169.36, 166.01, 164.86, 139.43, 133.46, 132.67, 122.82, 120.93, 118.35, 65.64, 55.31, 46.42, 36.82, 31.30, 30.22, 8.09 ppm. **^{195}Pt NMR** (500 MHz, D_2O): -1608 ppm. **HRMS** (MeOH/D_2O): 462.57374 [**2.Lu**+2H]$^{++}$.

2.Eu. Yield: 18%. **^{1}H NMR**(400MHz,D_2O): 35.42(s), 33.63(s), 30.02(s), 29.14(s), 28.33(s), 13.81-12.29(br), 8.08(s), 6.97(s), -1.59(s), -3.46(s), -4.06(s), -4.60(s), -7.63(s), -9.28(s),-9.47(s), -11.47(s), -11.80 (s), -12.26(s), -13.57(s), -13.89(s), -14.17(s), -15.84(s), -17.20(s)ppm. Only resolved peaks outside the 0 to 5 ppm region are reported. **HRMS** (MeOH): 451.0625 [**2.Eu**+2H]$^{++}$. UV-Vis (H2O): λ_{max} 345 nm.

2.Gd. Yield: 26%. **HRMS** (MeOH): 453.5641 [**2.Gd**+H+H]$^{++}$. UV-Vis (H_2O): λ_{max} 345 nm. UV-Vis (H_2O): λ_{max} 345 nm, **ICP-MS** Pt: Gd = 0.97:1 (Std Dev: 0.07).

General procedure for the synthesis of salicylic acidDO3A lanthanide-platinum(IV) complexes (3)

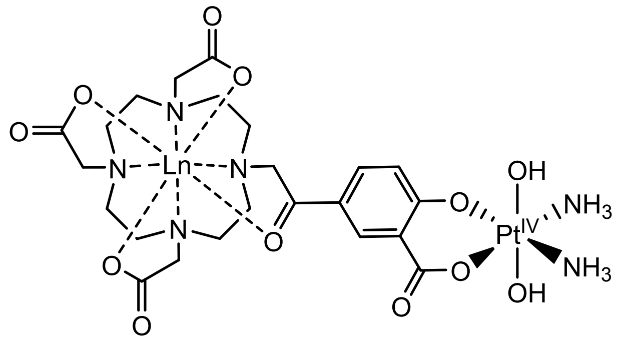

Figure S6. Compound **3.Ln**

To a solution of 0.02 mmol **2** in 1ml H2O, 80µL H_2O_2(30%wt) solution was added dropwise. The solution was stirred at room temperature overnight.
3.Eu. Yield: 60%. **^{1}H NMR**(400MHz,D_2O): 35.08(s), 33.35(s), 29.10(s), 28.04(s), 27.18(s), 15.23-10.05(br), 8.86(s), 8.25(s), 7.41(s), -1.74(s), -3.77(s), -4.37(s), -4.59(s), -7.52(s), -8.98(s) -9.70(s), -10.69(s), -11.40(s), -11.74(s), -12.74(s), -13.14(s),-13.58(s), -14.41(s), -15.77(s), -17.43(s) ppm. Only resolved peaks outside the 0 to 5 ppm region are reported. **HRMS** (MeOH): 935.1234 [**3.Eu**+H]$^+$. UV-Vis (H2O): λ_{max} 330 nm.

3.Gd. Yield: 58%. **HRMS** (MeOH): 942.12747 [**3.Gd**+H]$^+$. UV-Vis (H2O): λ_{max} 325 nm. UV-Vis (H_2O): λ_{max} 325 nm, **ICP-MS** Pt: Gd = 1.07:1 (Std Dev: 0.15).

General procedure for the synthesis of salicylic acidDO3A lanthanide-platinum(IV) bis octanoic acid complexes (4)

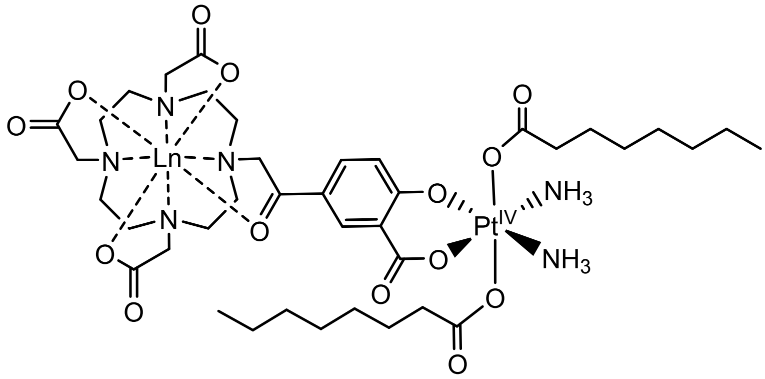

Figure S7. Compound **4.Ln**

3 (0.08 mmol) and pyridine (4 equiv.) was added to 3 ml acetone, a solution of n-octanoyl chloride (2.5 equivalent) in 2ml of acetone was added dropwise (over 15min). The reaction mixture was stirred overnight at room temperature. Solvent was reduced under reduced pressure and the crude product was purified by HPLC. **4.Gd. HRMS** (MeOH): 1192.3359 [**Gd.8**+H]$^+$. UV-Vis (H2O): λ_{max} 324 nm. **ICP-MS** Pt: Gd = 1.08:1 (Std Dev: 0.07).

Table S1. Elemental concentrations of Pt and Gd in complexes **1** – **4.Gd** determined over 5 replicates (R1 - R5).

	**Total Pt concentration (mM)**					**Total Gd concentration (mM)**				
	**Sample**					**Sample**				
replicate	Control	**1.Gd (1 mM)**	**2.Gd (0.5 mM)**	**3.Gd (0.5 mM)**	**4.Gd (1 mM)**	Control	**1.Gd (1 mM)**	**2.Gd (0.5 mM)**	**3.Gd (0.5 mM)**	**4.Gd (1 mM)**
R1	0.0	0.0	0.5	0.4	0.7	0.0	1.0	0.6	0.4	0.7
R2	0.0	0.0	0.5	0.4	0.9	0.0	1.0	0.5	0.4	0.8
R3	0.0	0.0	0.5	0.4	0.9	0.0	1.0	0.5	0.4	0.8
R4	0.0	0.0	0.5	0.4	0.8	0.0	1.1	0.5	0.4	0.8
R5	0.0	0.0	0.5	0.4	0.9	0.0	1.1	0.5	0.3	0.8

Cell culture for NMR In-cell diffusion

KNS42 cells were grown in DMEM (ThermoFisher, 61965026) supplemented with 10% foetal bovine serum (Sigma, F7524), and incubated at 37°C, 5% CO_2. Cell counting and viability were determined by Countess II automated cell counter (Invitrogen), using a 0.4% trypan blue solution.

Cell cytotoxicity testing:

To determine the cytotoxicity of each compound, KNS42 cells were prepared as described and exposed to serial dilutions of test compounds. In detail: 50 µL containing 5 x 10^3 KNS42 cells were seeded per well in white-walled, ½ area 96-well plates (Greiner, 655088). The following day the culture media was removed and replaced with 50 µL of compound containing media. Compounds were diluted in DMEM media to create a 10 point, ½ log serial dilution range from 1 mM to 0.0316 mM. Mock-treated and cell-free control wells were included for normalisation. Compound treated plates were incubated for 1.5 h, 24 h, 48 h and 72 h; after which 50 µL of CellTitre Glo (Promega, G7573) was added per well. Luminescence per well was determined using the BMG ClarioStar. Luminescence values were normalised by subtracting the value of the media only (mock-treated) wells. Cell viability per well was determined as the percentage of the maximal luminescence observed in the control (untreated) wells. IC_{50} values were calculated in Graph Pad Prism. 3 independent replicates were carried out for each compound.

In-Cell NMR:

KNS42 cells were grown in T-75 cm^2 flasks to a confluence not exceeding 80%. Cells were exposed to compounds at the highest tolerated dose determined by cytotoxicity assay at 1.5 h. After 1 h of exposure, the compound containing media was removed and cells were washed with 2 mL HBSS. Cell pellets were resuspended to a final concentration of $6e^7$/mL in drug containing media and placed on ice. Following NMR experiments, cell volumes were recovered, and the cell viability determined by trypan blue exclusion.

Statistical Analysis:

Statistical significance was determined by unpaired, two tailed *t*-test in graph pad prism. For all experiments, unless otherwise stated, 3 independent replicates were run.

NMR spectra

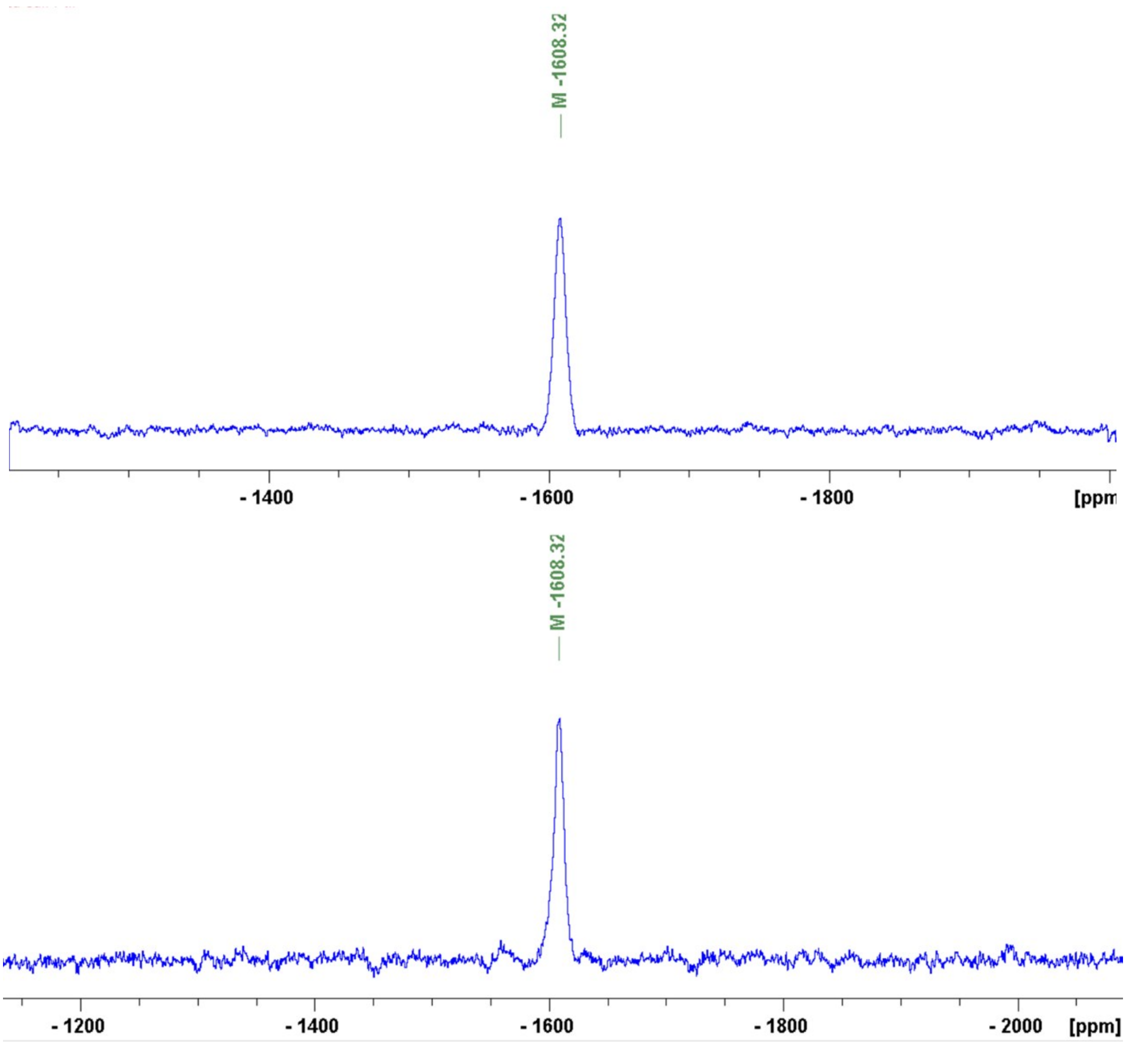

Figure S8. ^{195}Pt NMR spectrum (108 MHz, D_2O) of **2.Lu** (top) **and 2.Eu** (bottom).

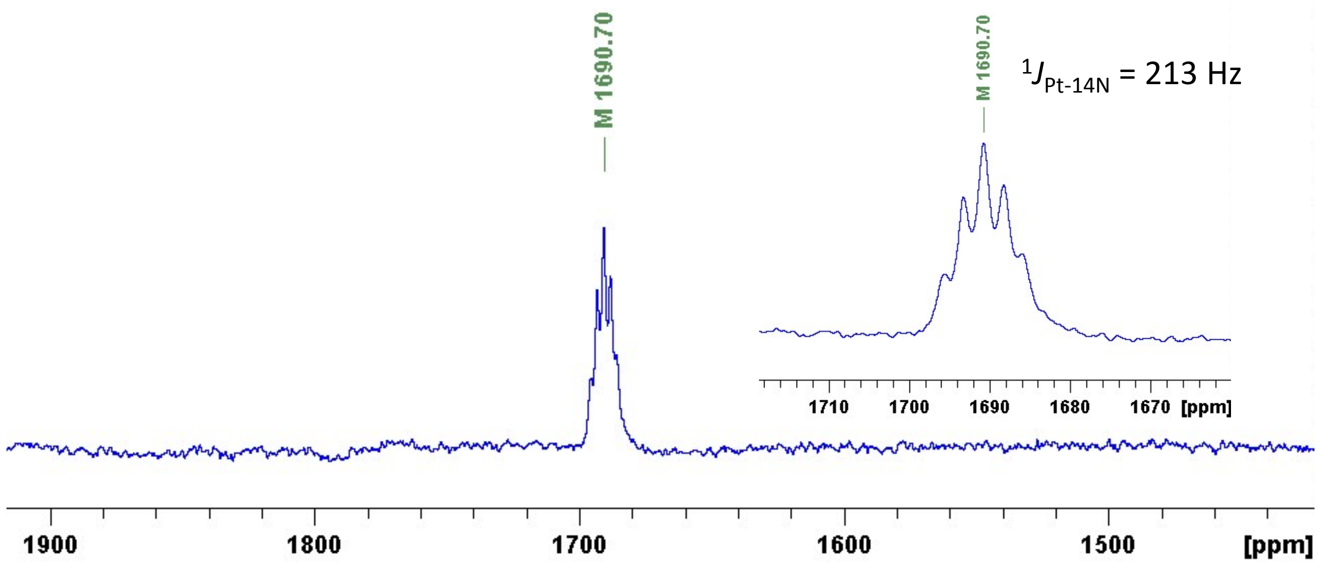

Figure S9. ^{195}Pt NMR spectrum (108 MHz, D_2O) of **3.Lu.**

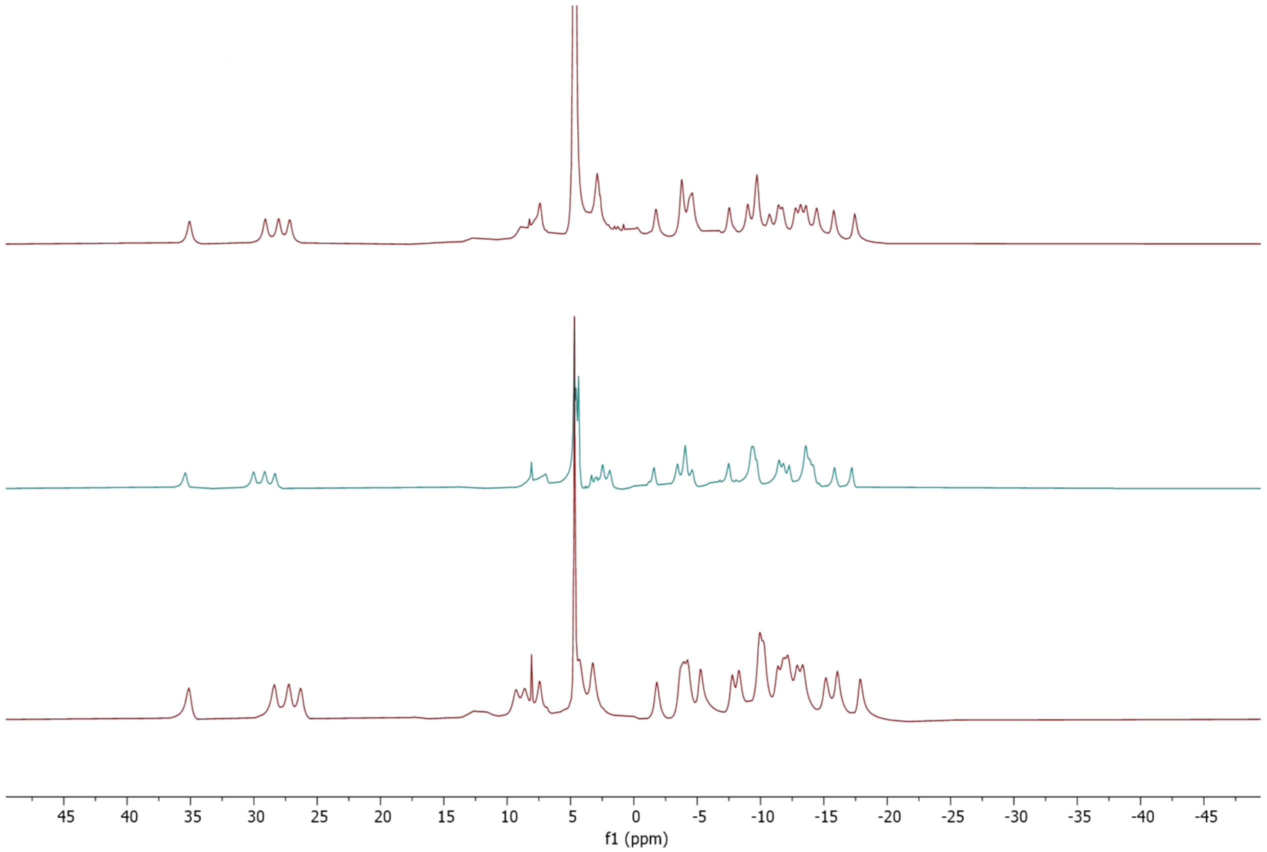

Figure S10. ^{1}H NMR spectra of **3.Eu** (top), **2.Eu** (middle) and **4.Eu** (bottom)

HRMS

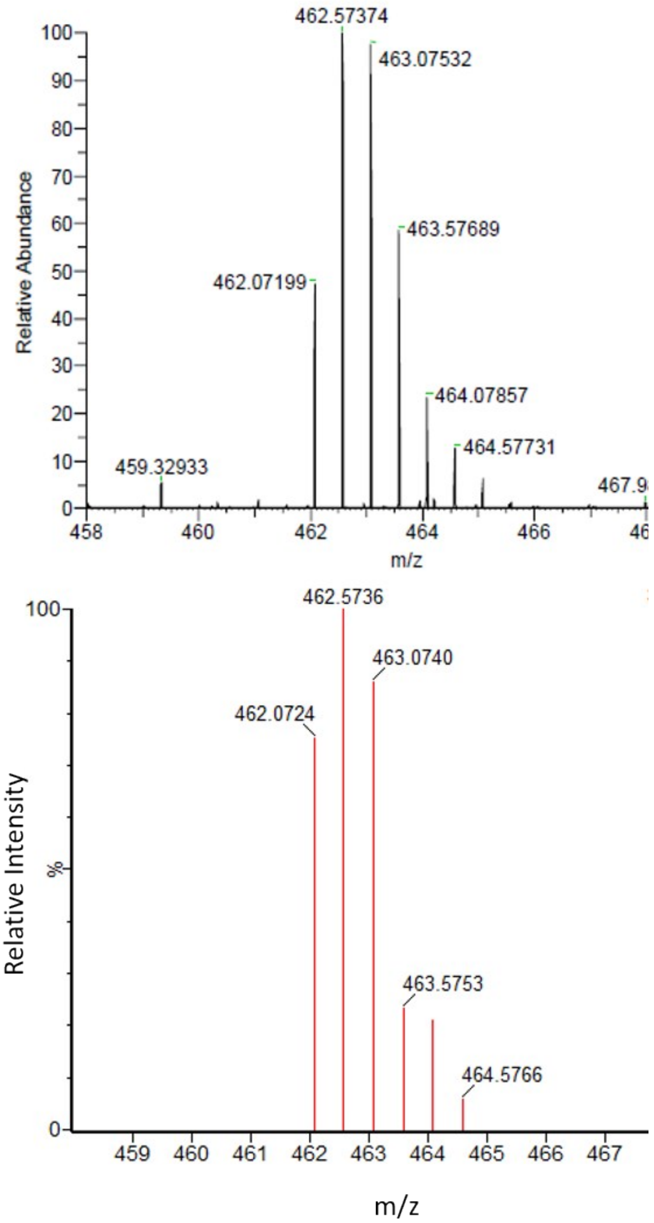

Figure S11. HRMS of [**2.Lu** +2H]$^{2+}$ (462.5737 *m/z*) in MeOH Molecular formula: $C_{23}H_{35}LuN_6O_{10}Pt$. Top: measured spectrum, bottom: predicted spectrum. Mass error: 0.3 ppm.

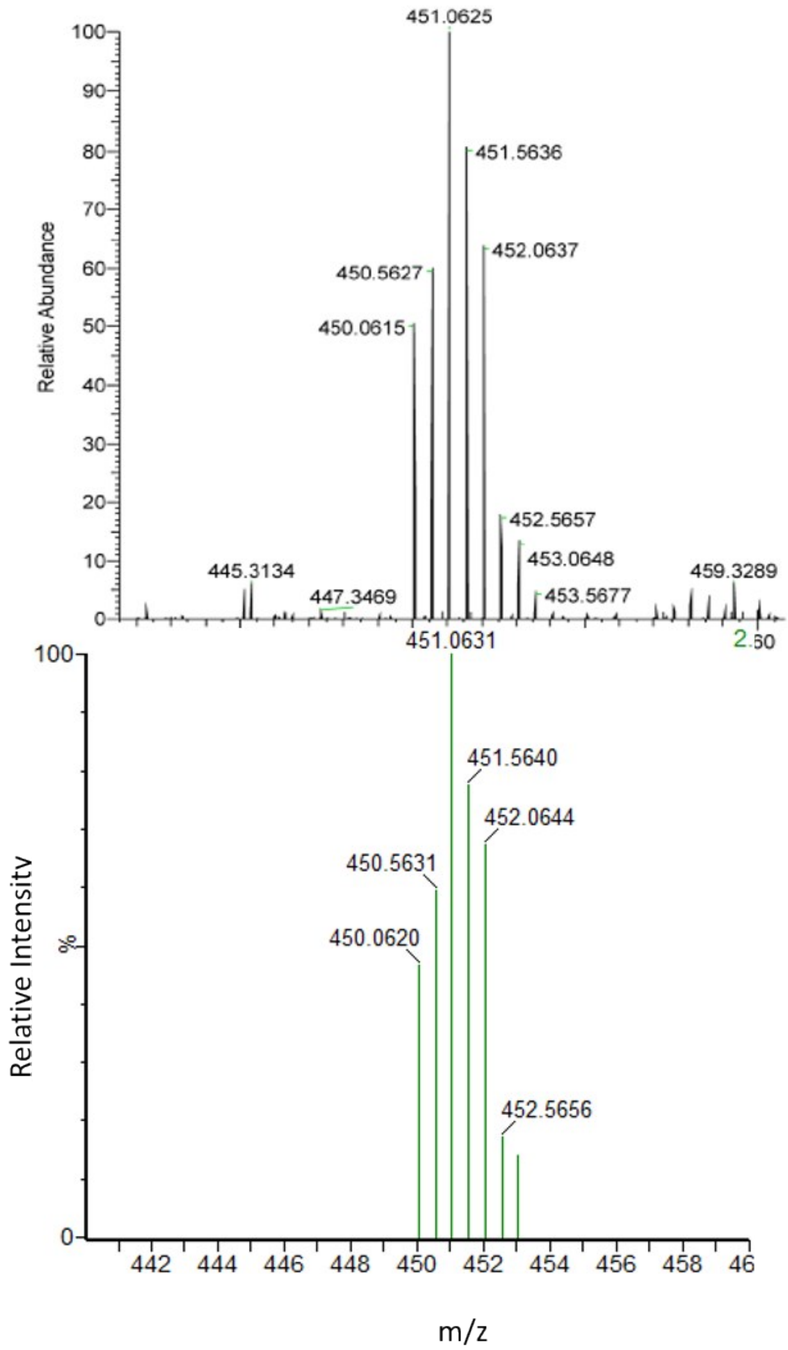

Figure S12. HRMS of [**2.Eu** +2H]$^{2+}$ in MeOH. Molecular formula: $C_{23}H_{35}EuN_6O_{10}Pt$. Top: measured spectrum, bottom: predicted spectrum. Mass error: 1.3 ppm.

HPLC

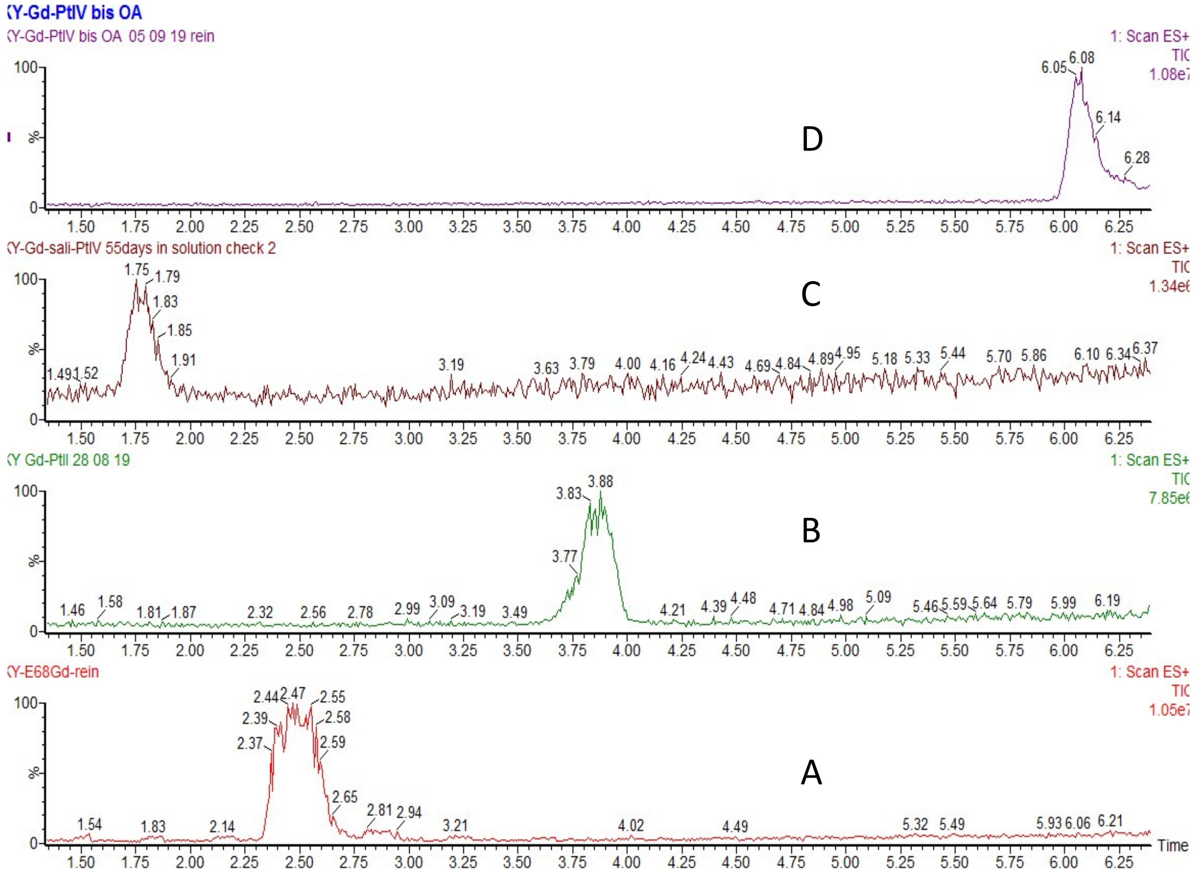

Figure S13. HPLC traces of A) **1.Gd**, t_R = 2.47; B) **2.Gd**, t_R = 3.88; C) **3.Gd**, t_R = 1.75 and D) **4.Gd,** t_R = 6.08. Ln-Pt^{II} and Ln-Pt^{IV} complexes are stable in solution at 4°C for at least 50 d, judged by reinjecting the complexes in the HPLC.

Single Crystal X-ray Diffraction Studies

Single crystal X-ray structure determination of 2.Eu: Low temperature single crystal X-ray diffraction data were collected using a (Rigaku) Oxford Diffraction SuperNova A diffractometer. Raw frame data were reduced using CrysAlisPro and the structure was solved from the integrated intensities with charge-flipping using 'Superflip'.[3] The structure was refined using full-matrix least squares on F^2 using the CRYSTALS suite.[4–6] Crystallographic data have been deposited with the Cambridge Crystallographic Data Centre (CCDC: 1960280) and can be obtained via www.ccdc.cam.ac.uk/data_request/cif.

Crystal Data

Table S2. Crystal data and structure refinement for **2.Eu**.

Empirical formula	$C_{23}H_{60}EuN_6O_{23.5}Pt$
Formula weight	1143.81
Temperature	150 K
Wavelength	1.54184 Å
Crystal system	Orthorhombic
Space group	P b c n
Unit cell dimensions	a = 22.1561(1) Å α= 90°.<br>b = 18.9237(1) Å β= 90°.<br>c = 19.3441(1) Å γ = 90°.
Volume	8110.50(7) $Å^3$
Z	8
Density (calculated)	1.873 Mg/m^3
Absorption coefficient	18.119 mm^{-1}
F(000)	4552
Crystal size	0.21 x 0.15 x 0.14 mm^3
Theta range for data collection	3.829 to 76.242°.
Index ranges	-27<=h<=27, -23<=k<=20, -23<=l<=23
Reflections collected	47882
Independent reflections	8432 [R(int) = 0.036]
Completeness to theta = 74.717°	99.7 %
Absorption correction	Sphere
Max. and min. transmission	0.04823 and 0.00242
Refinement method	Full-matrix least-squares on F^2
Data / restraints / parameters	8403 / 0 / 517
Goodness-of-fit on F2	0.9995
Final R indices [I>2sigma(I)]	R1 = 0.0853, wR2 = 0.2000
R indices (all data)	R1 = 0.0869, wR2 = 0.2057
Largest diff. peak and hole	4.76 and -1.11 e.$Å^{-3}$

UV spectra

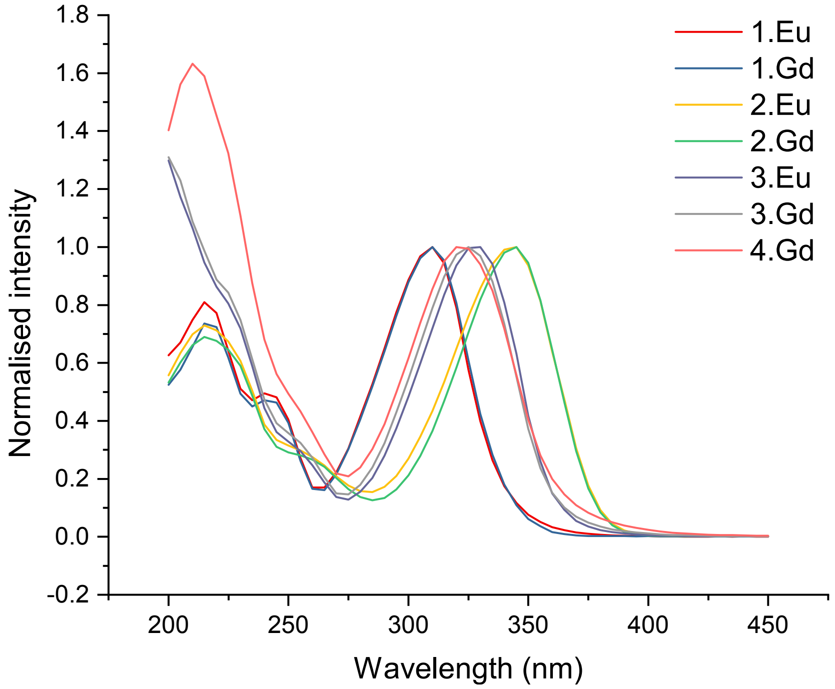

Figure S14. UV-Vis absorption of Eu and Gd complexes, **1.Ln**: 309nm, **4.Gd**: 324nm, **3.Gd**: 325nm, **3.Eu**: 330nm, **2.Ln**: 345 nm

Excitation and Emission Spectra

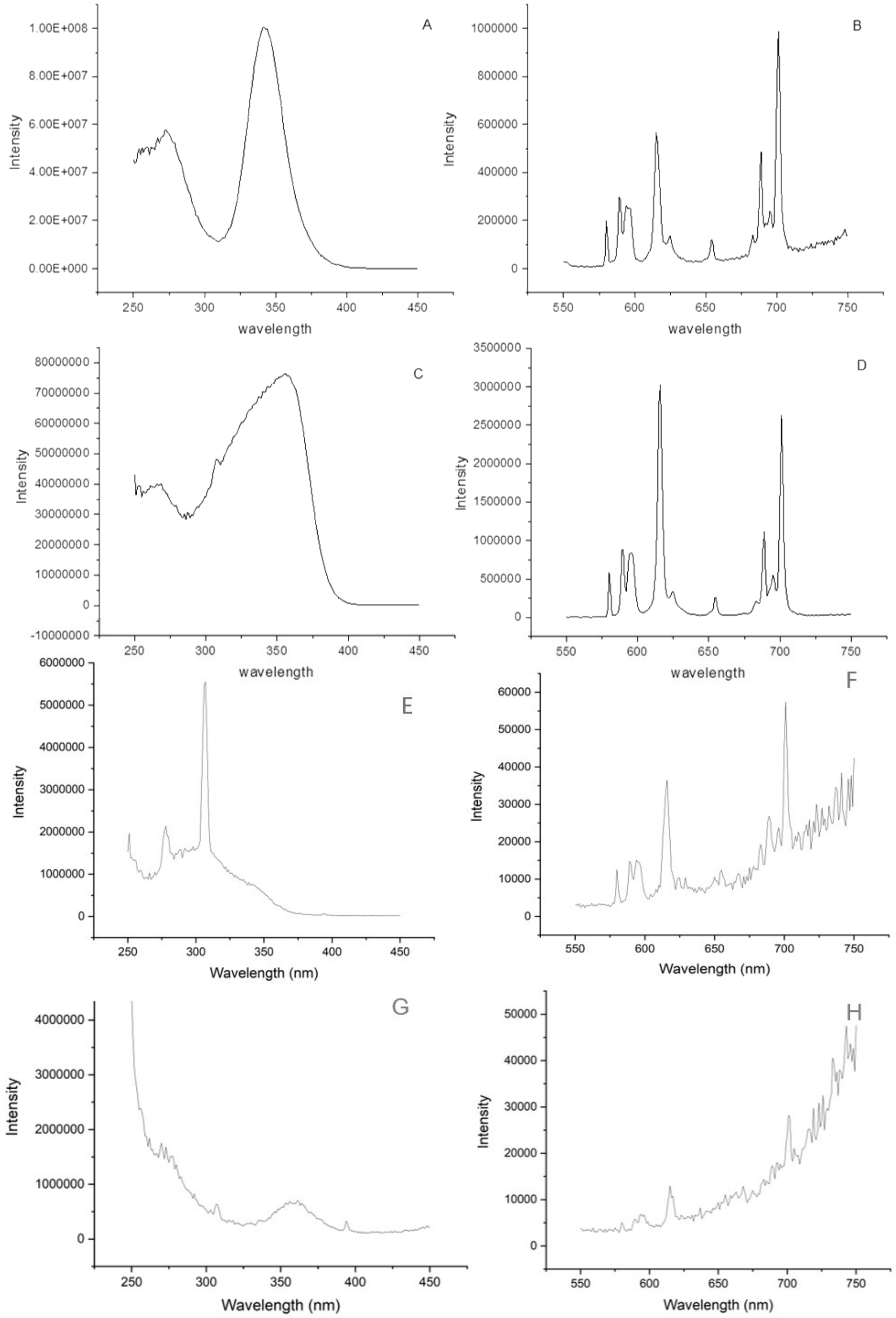

Figure S15. A): Excitation spectrum of **1.Eu** (λ_{em} = 616 nm), B) Emission spectrum of **1.Eu** (λ_{ex} = 347 nm), C): Excitation spectrum of **2.Eu** (λ_{em} = 616 nm), D) Emission spectrum of **2.Eu** (λ_{ex} = 345 nm). E): Excitation spectrum of **3.Eu** (λ_{em} = 616 nm), and F) Emission spectrum of **3.Eu**(λ_{ex} = 345 nm). G): Excitation spectrum of **4.Eu** (λ_{em} = 616 nm), and H) Emission spectrum of **4.Eu**(λ_{ex} = 345 nm). All samples are equimolar 10^{-4} M in H_2O. * 313 nm artefact.

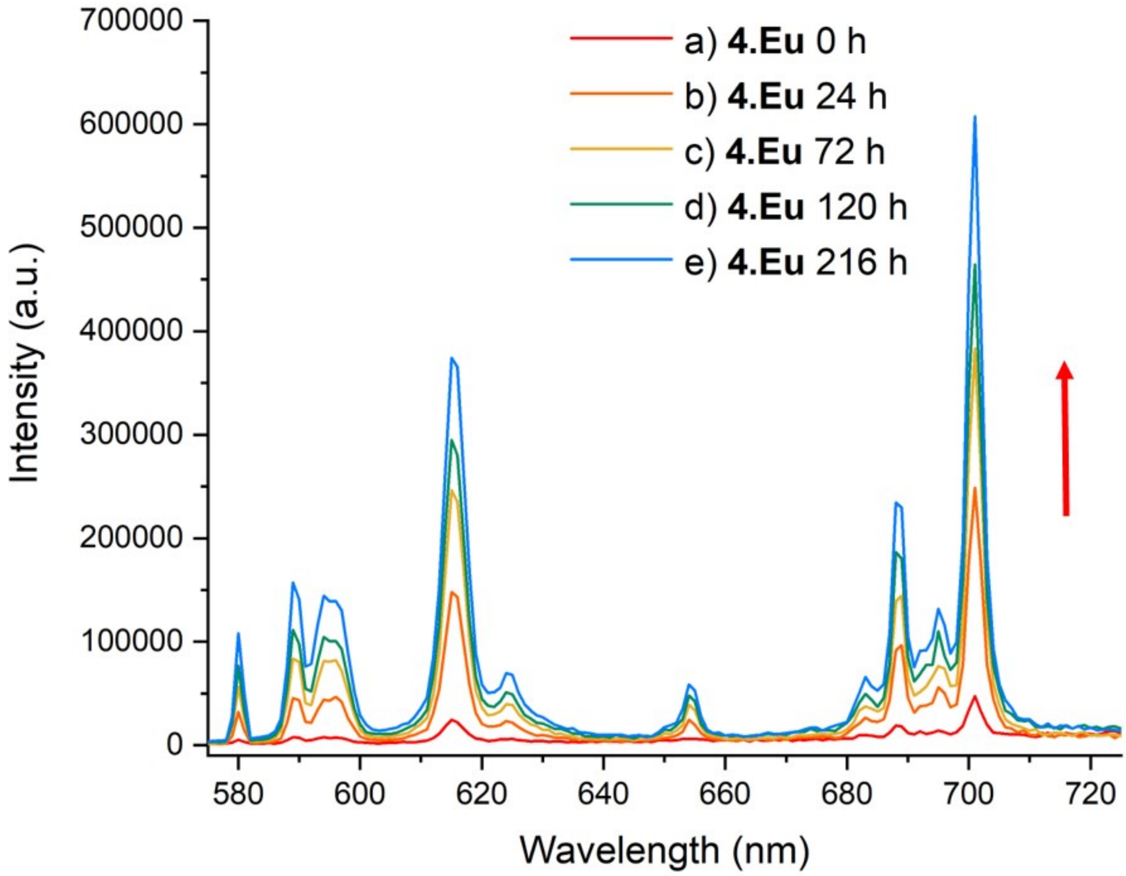

Figure S16. Luminescence spectrum of **4.Eu**; λ_{ex} = 330 nm, emission slit = 1 nm. a) before addition, b) 24 h after addition of excess (20-fold) ascorbic acid (AA) and c) 72 h after addition of AA, d) 120 h after addition of AA, e) 216 h after addition of AA. The red arrow shows that as time increases the intensity of emission increases.

Lifetimes

Table S3. Lifetime values of the excited states of the luminescent Eu-Sali acid complex **1.Eu** and Eu-Pt^{II} complex **2.Eu**. The measurement of the lifetimes of luminescence in water and D_2O allowed the determination of the hydration number using the modified Horrocks' equation.[7]

Complex	λ_{ex}/nm	λ_{em}/nm	τ_{H2O}/µs	τ_{D2O}/µs	q
**1.Eu**	347	616	657	2131	0.96
**2.Eu**	345	616	609	1869	1.03

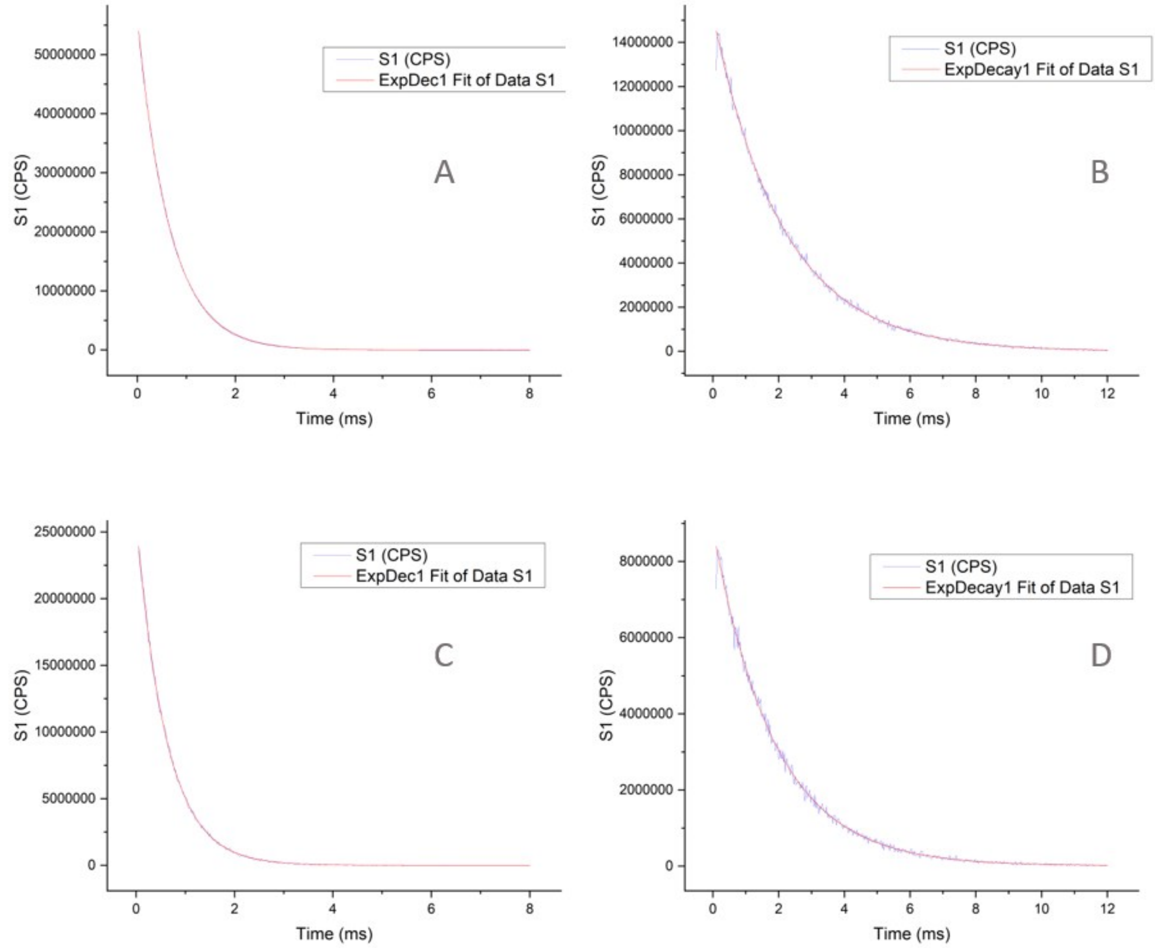

Figure 17. Lifetimes for the Eu complexes: A): Eu-Sali acid complex **1.Eu** in H_2O, τ = 0.66 ms. B): Eu-Sali acid complex **1.Eu** in D_2O, τ = 2.13 ms**.** C): Eu-Pt^{II} complex **2.Eu** in H_2O, τ = 0.61 ms**.** D): Eu-Pt^{II} complex **2.Eu** in D_2O, τ = 1.87 ms**.**

Cytotoxicity Experiments

Table S4. KNS42 compound toxicity. KNS42 cells were exposed to ½ log serial dilutions of test compounds for 1.5, 24, 48 and 72 hours. IC_{50} values were determined in Graphpad prism using a 3 parameter log inhibitor vs. concentration curve fit. 3 independent replicates (n=2) were performed for all compounds. Mean and standard deviation of IC_{50} values are shown. *P* values were calculated in GraphPad Prism.

KNS42 Compound Cytotoxicity						
Mean IC_{50} ± Standard Deviation						
	**1.5 h**		**IC_{50} Significant Difference (*P* value)**			
**Treatment**	**Mean IC_{50}**	**St.Dev**	**1.Gd**	**2.Gd**	**3.Gd**	**4.Gd**
**1.Gd**	>1000 µM	0 µM	n/a	0.0001	<0.0001	n/a*
**2.Gd**	637.9 µM	43.27 µM	0.0001	n/a	<0.0001	0.0001
**3.Gd**	227.6 µM	15.59 µM	<0.0001	<0.0001	n/a	<0.0001
**4.Gd**	>1000 µM	0 µM	n/a*	0.0001	<0.0001	n/a
	**24 h**		**IC_{50} Significant Difference (*P* value)**			
**1.Gd**	>1000 µM	0 µM	n/a	<0.0001	<0.0001	n/a*
**2.Gd**	316.7 µM	51.75 µM	<0.0001	n/a	0.0031	<0.0001
**3.Gd**	96.48 µM	25.77 µM	<0.0001	0.0031	n/a	<0.0001
**4.Gd**	>1000 µM	0 µM	n/a*	<0.0001	<0.0001	n/a
	**48 hr**		**IC_{50} Significant Difference (*P* value)**			
**1.Gd**	>1000 µM	0 µM	n/a	<0.0001	<0.0001	0.0027
**2.Gd**	75.28 µM	8.79 µM	<0.0001	n/a	0.0584	<0.0001
**3.Gd**	91.42 µM	5.98 µM	<0.0001	0.0584	n/a	<0.0001
**4.Gd**	747.5 µM	0 µM	0.0027	<0.0001	<0.0001	n/a
	**72 hr**		**IC_{50} Significant Difference (*P* value)**			
**1.Gd**	>1000 µM	0 µM	n/a	<0.0001	<0.0001	<0.0001
**2.Gd**	75.74 µM	14.11 µM	<0.0001	n/a	0.1482	0.0013
**3.Gd**	53.24 µM	17.99 µM	<0.0001	0.1482	n/a	0.001
**4.Gd**	142.8 µM	2.48 µM	<0.0001	0.0013	0.001	n/a

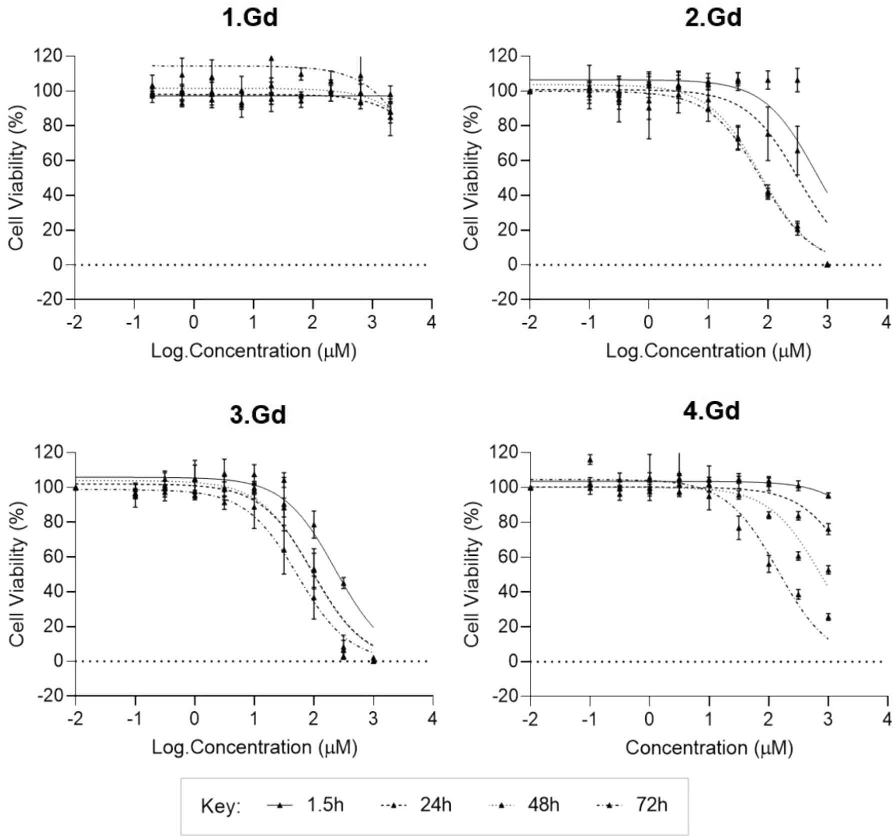

Figure S18. KNS42 compound toxicity. KNS42 cells were exposed to ½ log serial dilutions of test compounds for 1.5, 24, 48 and 72 h. IC_{50} values were determined in Graphpad prism using a 3 parameter log inibitor *vs.* concentration curve fit. Three independent replicates were performed.

References

[1] G. Karunanithy, R. J. Wheeler, L. R. Tear, N. J. Farrer, S. Faulkner, A. J. Baldwin, *J. Magn. Reson.* **2019**, *302*, 1–13.

[2] A. Dadabhoy, S. Faulkner, P. G. Sammes, *J. Chem. Soc. Perkin Trans. 2* **2002**, 348–357.

[3] L. Palatinus, G. Chapuis, *J. Appl. Crystallogr.* **2007**, *40*, 786–790.

[4] P. W. Betteridge, J. R. Carruthers, R. I. Cooper, K. Prout, D. J. Watkin, *J. Appl. Crystallogr.* **2003**, *36*, 1487–1487.

[5] R. I. Cooper, A. L. Thompson, D. J. Watkin, *J. Appl. Crystallogr.* **2010**, *43*, 1100–1107.

[6] P. Parois, R. I. Cooper, A. L. Thompson, *Chem. Cent. J.* **2015**, *9*, 1–14.

[7] A. Beeby, I. M. Clarkson, R. S. Dickins, S. Faulkner, D. Parker, L. Royle, A. S. de Sousa, J. A. G. Williams, M. Woods, *J. Chem. Soc. Perkin Trans. 2* **1999**, *2*, 493–504.
